# Plasma proteome profiling of healthy individuals across the life span in a Sicilian cohort with long‐lived individuals

**DOI:** 10.1111/acel.13684

**Published:** 2022-08-06

**Authors:** Valentina Siino, Ashfaq Ali, Giulia Accardi, Anna Aiello, Mattia E. Ligotti, Sergio Mosquim Junior, Giuseppina Candore, Calogero Caruso, Fredrik Levander, Sonya Vasto

**Affiliations:** ^1^ Department of Immunotechnology Lund University Lund Sweden; ^2^ National Bioinformatics Infrastructure Sweden (NBIS), Science for Life Laboratory, Department of Immunotechnology Lund University Lund Sweden; ^3^ Laboratory of Immunopathology and Immunosenescence, Department of Biomedicine, Neurosciences and Advanced Diagnostics University of Palermo Palermo Italy; ^4^ Department of Biological, Chemical and Pharmaceutical Sciences and Technologies University of Palermo Palermo Italy; ^5^ Euro‐Mediterranean Institutes of Science and Technology (IEMEST) Palermo Italy

**Keywords:** aging, longevity, plasma proteome

## Abstract

The study of healthy human aging is important for shedding light on the molecular mechanisms behind aging to promote well‐being and to possibly predict and/or avoid the development of age‐related disorders such as atherosclerosis and diabetes. Herein, we have employed an untargeted mass spectrometry‐based approach to study age‐related protein changes in a healthy Sicilian plasma cohort including long‐lived individuals. This approach confirmed some of the previously known proteins correlated with age including fibulin‐1, dystroglycan, and gamma‐glutamyl hydrolase. Furthermore, our findings include novel proteins that correlate with age and/or with location and uric acid, which could represent a unique signature for healthy aging.

## Graphical abstract

Mass spectrometry proteomics was used to detect age‐related plasma proteome changes across the lifespan in a cohort including long‐lived individuals. The approach allowed for detection of novel and already known protein level changes related to aging.

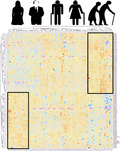

**Abbreviations**
ACN: acetonitrile
AMBIC: ammonium bicarbonate
FA: formic acid
HILIC: hydrophilic interaction liquid chromatography
LC–MS/MS: liquid chromatography–tandem mass spectrometry
NH4Ac: ammonium acetate
PC: principal component
SDS: sodium dodecyl sulfate
TRIS: tris[hydroxymethyl] aminoethane

## 1 INTRODUCTION

Aging, defined as a time‐dependent functional decline of living organisms (López‐Otín et al., 2013), is characterized by a progressive deterioration of physiological functions, often leading to development of age‐related diseases such as atherosclerosis, neurodegenerative disorders and diabetes (Vasto et al., 2007).

Several factors have been characterized as risks for the development of age‐related diseases such as genomic predispositions, telomeric and epigenetic alterations, mitochondrial dysfunction, and cellular senescence (López‐Otín et al., 2013). Also, chronic systemic inflammation might lead to higher risk of developing cardiovascular diseases, both representing a major cause of death in people older than 65 years. Inflammation is largely related to senescent cells, which increase in older tissues, as these cells produce inflammatory mediators, acquiring the senescence‐associated secretory phenotype (Ferrucci & Fabbri, 2018).

Long‐lived individuals (LLIs) represent a good model of healthy aging since, over the years, they have either escaped, delayed, or survived to age‐related diseases and show good health. Investigating aging mechanisms and, even more, how LLIs have developed a healthy aging process, is of great interest to unravel potential biomarkers that could prolong human life span and/or promote healthy aging (Accardi, Aiello, et al., 2019).

Even though plasma proteomics is very challenging (Anderson & Anderson, 2002), the development of high‐throughput approaches has increased the potential for studying circulating plasma proteins. To date, collecting plasma is a feasible and low‐invasive procedure, and the use of proteomics to analyze plasma proteins could contribute to the identification of unique protein signatures in the population of study (herein a healthy cohort of younger and older individuals). A growing number of recent studies have shown the feasibility of plasma proteomics to tackle changes as function of aging (Lehallier et al., 2019; Moaddel et al., 2021).

The aim of the present research was to analyze the plasma proteome in different cohorts of the Sicilian population, from young to ultracentenarians, to identify protein patterns across the life span that are specifically related to healthy aging and longevity. Thus, we analyzed a well‐characterized homogeneous sample of 86 people from Western Sicily, an Italian island in the center of Mediterranean Sea. This population is part of a larger sample cohort where we have already collected anamnestic data and performed hematological, hematochemical, molecular, and oxidative stress analyses, adopting a multidimensional analysis approach (Aiello et al., 2021). The collection of these anamnestic data allowed for the correlation of these parameters with protein quantities.

To reduce sample preparation variation between samples, we used a high‐throughput automated protein digestion approach and combined it with a data‐independent acquisition (DIA) liquid chromatography–tandem mass spectrometry (LC–MS/MS) method to deepen protein coverage. Using this method, in combination with statistical analysis, we could confirm some of the findings from other aging studies, and we were able to further identify new proteins that could play an important role in healthy aging.

## 2 RESULTS

In the present study, the primary aim was to use an untargeted proteomic approach for analysis of plasma to reveal proteins that could be associated with healthy aging and longevity. Specifically, we used an automated high‐throughput plasma protein preparation method and combined it with DIA LC–MS/MS to identify differentially expressed proteins in plasma between different age groups from a healthy Sicilian cohort. We further aimed to use the dataset to investigate whether plasma proteome differences correlated to other anthropometric factors could be revealed.

### 2.1 Plasma aging proteome overview

**FIGURE 1:**
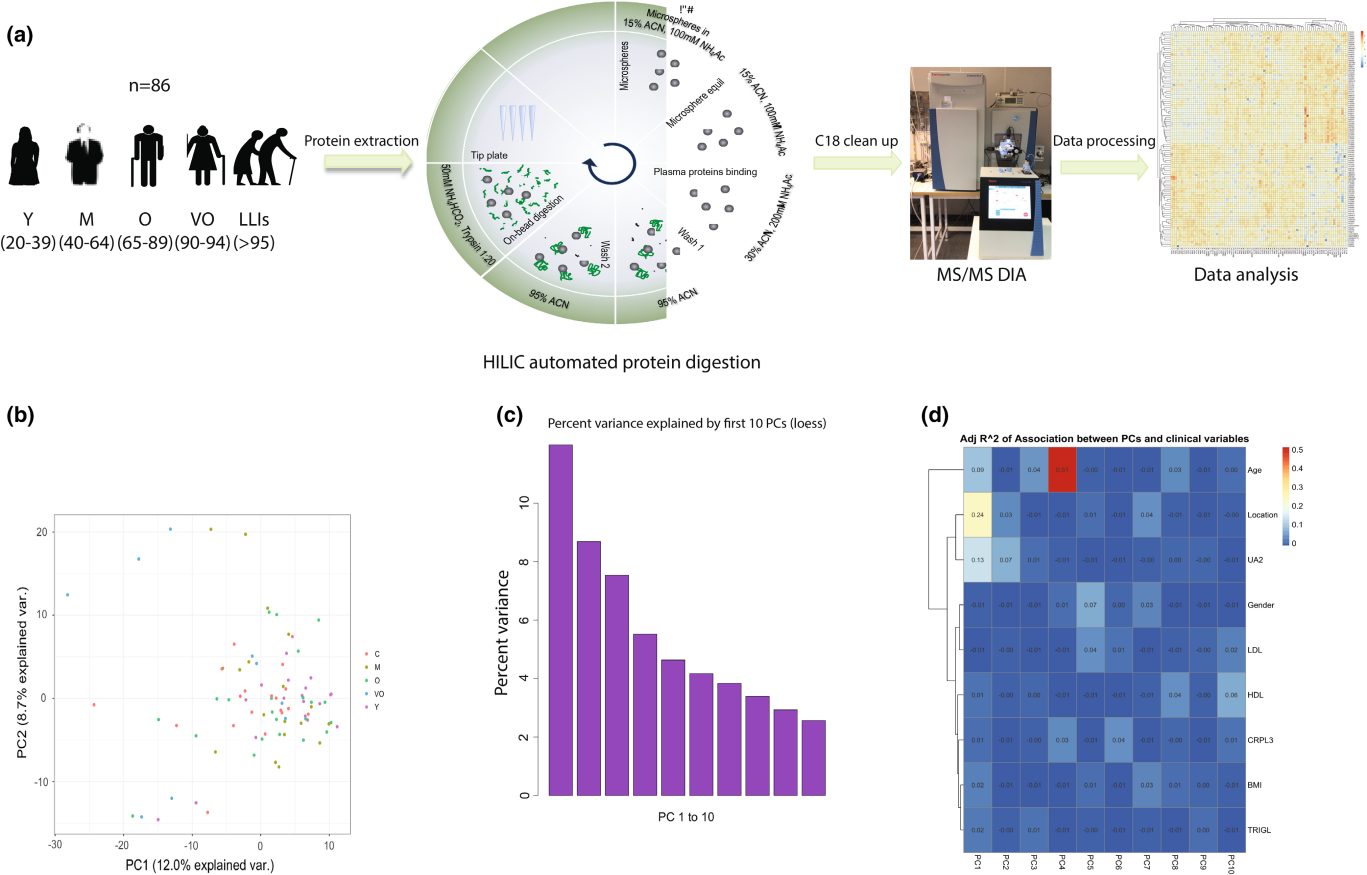
(a) Schematic overview of the analysis workflow. Individuals were divided based on their age range in young (Y = 20–39), middle age (M = 40–64), old (O = 65–89), very old (VO = 90–94), and long‐lived individuals (LLIs > 95–111). (b) Principal component analysis plot showing the distribution of each sample based on protein quantities. (c) Percent variance in the principal components, showing sample distribution in the first 10 principal components. (d) Correlation between the principal components and clinical parameters: age, location, uric acid (UA2), gender, LDL, HDL, CRPL3, BMI, and TRIGL. Age, location, and UA were the main parameters associated with protein distribution.

The current study included a total of 86 plasma samples from healthy men and women aged 22 to 111 years. A schematic overview of the samples and the workflow employed are displayed in Figure 1(a). Further, individuals' information regarding some biochemical parameters, as well as BMI, age and location (origin of the samples), is reported in Table S1.

Using an automated sample processing and a label‐free approach on undepleted plasma combined with data‐independent acquisition (DIA), a total of 435 proteins were identified and quantified. After inspection of the data, some proteins were removed due to more than 50% missing values across samples and the final protein list contained 410 proteins (Table S2).

Figure 1(b) displays the overall sample distribution in the principal component analysis (PCA) plot. Percent variance explained in the principal components (PCs), shows a good distribution of the data in the first 10 PCs (Figure 1c). Thus, the association between the 10 PCs and the clinical variables collected from each individual was further investigated. Results show that parameters such as age, location (towns or villages), uric acid (UA), and gender play a role in protein distribution, while biochemical factors such as LDL, HDL, and BMI do not significantly contribute to the distribution of proteins (Figure 1d).

### 2.2 Protein correlation with age

**FIGURE 2:**
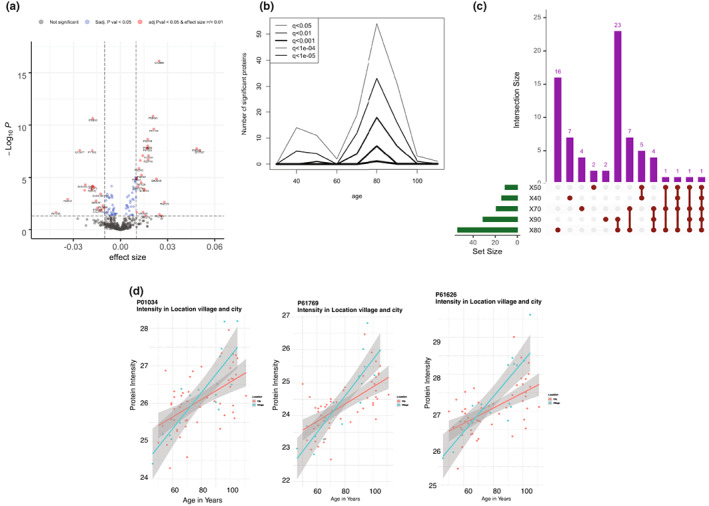
(a) Volcano plot depicting protein correlations with age. Red dots represent proteins that are significantly correlating with age at an adjusted *p* <0.05 and an effect size cutoff >/<0.01. Blue dots represent significant proteins based only on the adjusted *p* < 0.05. Grey dots are nonsignificant proteins, proteins that have not passed a statistical cutoff or an effect size cutoff. (b) DEswan analysis of proteins in 10‐year windows. Two major peaks of significant proteins at different q‐value cutoffs, one at 40 years old and another one at 80 years old, could be identified. (c) Upset plot depicting significant proteins in the 10‐year windows (X40, X50, X70, X80, and X90). The plot shows unique significant proteins for some windows (i.e., 16 unique significant proteins for X80), as well as some overlap between age windows. (d) Linear representation of three representative proteins and their intensity distribution, based on the location (red = towns, blue = villages): cystatin‐C (P01034, CYTC), beta‐2‐metaglobulin (P61769, B2MG), and lysozyme C (P61626, LYSC).

Based on the PC distribution, the correlation between proteins and age was further investigated. From this analysis, we identified 106 proteins that significantly (q‐value < 0.05) correlated with age (Figure S1). To further discriminate between up‐ and down‐regulated proteins, effect size was estimated using log2‐transformed protein intensity. Each year in age represents 0.01 increase in the log‐transformed protein intensity at this cutoff. Twenty age‐associated proteins were negatively regulated, while 27 were positively regulated as shown in a volcano plot (Figure 2a). Among this last group of proteins, fibulin‐1 (FBLN1), lysozyme C (LYZ), dystroglycan (DAG1), and gamma‐glutamyl hydrolase (GGH) have previously been reported as related to aging (Lehallier et al., 2019).

Most of the age‐associated proteins that are negatively correlated with age are immunoglobulins (IGHG2, IGHM, IGHV3‐72, IGKV1‐8, IGKV2‐28, IGLV2‐23, IGLV7‐43, IGLV7‐46) and two insulin‐like growth factor‐binding proteins (IGFBP‐3 and IGFBP‐5).

To further characterize proteome changes during individuals' life span, we used the differential expression‐sliding window analysis (DEswan) algorithm, which has been demonstrated to provide useful information about protein changes at particular stages of life (Lehallier et al., 2019). The algorithm employed was set to analyze protein levels within a window of 10 years. Using this approach, we were able to identify significant proteins changing mainly in two age waves with a peak at age 40 and then a consistent peak at age 80 (Figure 2b). The upset plot (Figure 2c) is also showing the number of significant proteins that are unique or shared between age waves. Most of the significant proteins in the age waves are unique to LLIs; in fact, the age range X80 comprises of 16 proteins, while X80 and X90 share 22 proteins (Table S3). Among these proteins, many of them are related to extracellular matrix (ECM) structure and functions (Table 1). The 7 proteins unique to age range 40 are related to the immune response: immunoglobulin mu heavy chain, immunoglobulin heavy constant mu, CD5 antigen‐like, cartilage oligomeric matrix protein, C‐reactive protein, L‐selectin, and lipopolysaccharide‐binding protein.

**TABLE 1:** DEswan analysis reveals that 21 proteins in the age waves are related to the extracellular matrix

Protein ID	Protein name	Gene name	Age wave
P02647	Apolipoprotein A‐1	APOA1	X80
P55290	Cadherin‐13	CDH13	X80
P54108	Cysteine‐rich secretory protein 3	CRISP3	X80
P00748	Coagulation factor XII	F12	X80
P02671	Fibrinogen alpha chain	FGA	X80
P02675	Fibrinogen beta chain	FGB	X80
P02679	Fibrinogen gamma chain	FGG	X80
P51884	Lumican	LUM	X80
P01011	Alpha‐1‐antichymotrypsin (Serpin A3)	SERPINA3	X90
Q14118	Dystroglycan	DAG1	Shared between X80 and X90
P00734	Prothrombin	F2	Shared between X80 and X90
P19827	Inter‐alpha‐trypsin inhibitor heavy chain H1	ITIH1	Shared between X80 and X90
P01008	Antithrombin‐III	SERPINC1	Shared between X80 and X90
P08294	Extracellular superoxide dismutase	SOD3	Shared between X80 and X90
Q15582	Transforming growth factor beta‐induced protein ig‐h3	TGFBI	Shared between X80 and X90
P22105	Tenascin‐X	TNXB	Shared between X80 and X90
P04004	Vitronectin	VTN	Shared between X80 and X90
Q14767	Latent‐transforming growth factor beta‐binding protein 2	LTBP2	Shared between X70 and X80
P04275	Von Willebrand factor	VWF	Shared between X70 and X80
P36222	Chitinase‐3‐like protein 1	CHI3L1	Shared between X70 and X80
P20774	Mimecan (Osteoglycin)	OGN	Shared between X70, X80, and X90

### 2.3 Protein correlation with location, gender, and uric acid

In the present dataset, most of the plasma samples were collected from individuals living in towns (Palermo or other towns of western Sicily), while some of the plasma from LLIs and their relatives were coming from villages in the Sicilian mountains (Madonie or Sicani mounts). Nine proteins correlate to the location at a set q‐value < 0.05 (Figure S2). Figure 2(d) shows the protein intensity (*y*‐axis) plotted against age (*x*‐axis) of 3 representative proteins related to the cellular protein metabolic process (B2MG, LYSC, and CYTC) and their distribution with regard to age and location (blue line = villages and red line = towns). The expression of these proteins is increasing with age, being more represented in the older population from the villages compared with the towns.

**FIGURE 3:**
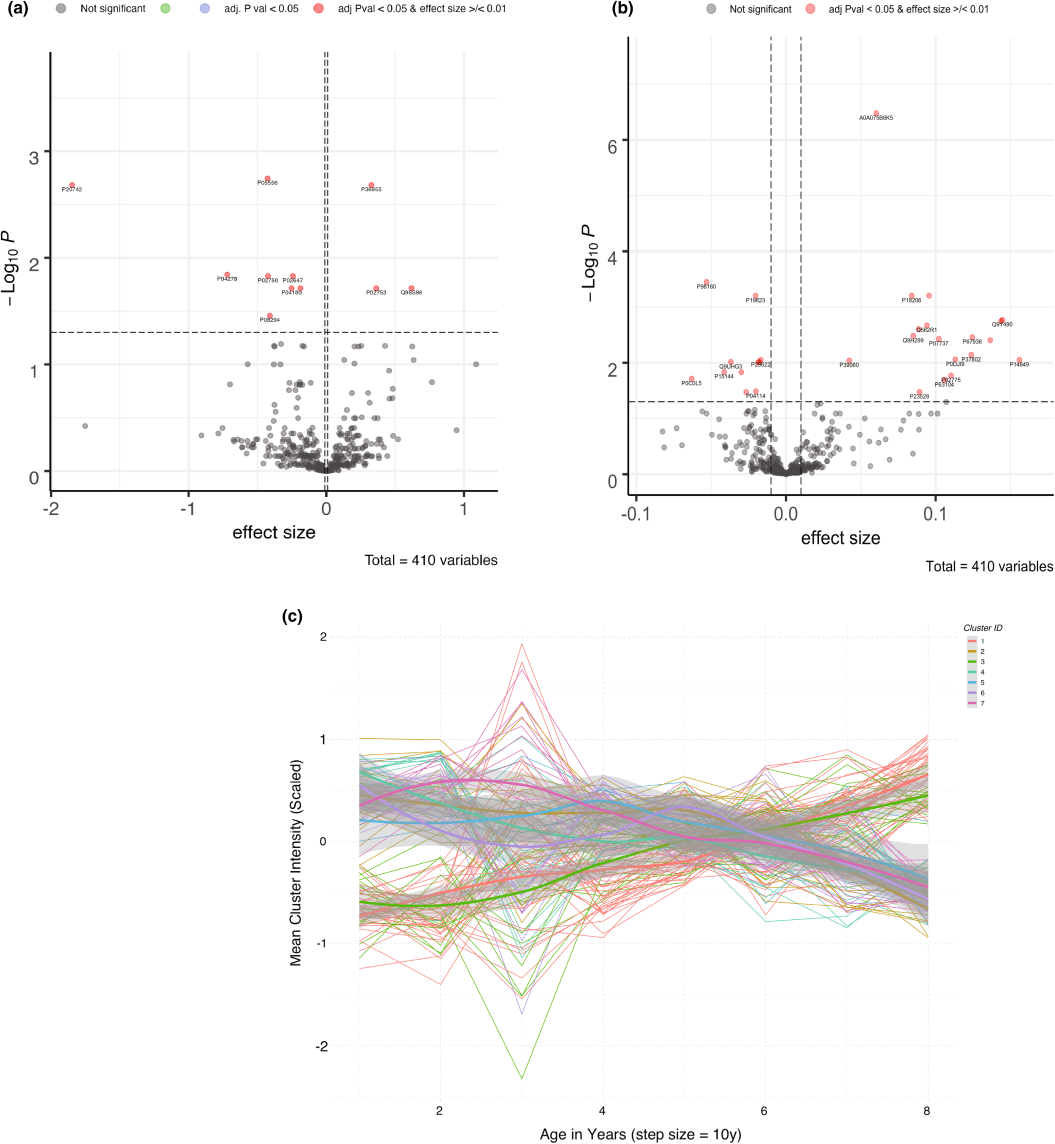
(a,b) Volcano plots showing gender‐related and uric acid‐related proteins, respectively. Significant proteins (adjusted *p* < 0.05 and effect size >/<0.01) are depicted in red. Grey dots represent the non‐significant proteins. (c) Plasma protein clusters and their trajectories plotted against age in years (*x*‐axis).

Furthermore, 11 proteins were significantly correlated to gender (Figure 3a) and, as expected, 2 proteins are hormonal proteins (SHBG and PZP).

In the present cohort, 29 proteins are significantly correlated to uric acid (UA): 18 of them are positively correlated to UA, while 11 proteins are negatively correlated, as shown in Figure 3(b). Of the 18 proteins positively correlated to UA, 9 of them are part of the cytoskeleton machinery such as ACT, CFL1, FLN‐A, PFN1, TLN1, TPM4 and VCL.

### 2.4 Cluster of significant proteins and their trajectories

Normalized significant proteins were divided into clusters with an age step size of 10 years. As depicted in Figure 3(c), the first 7 clusters show high intensity and were further investigated. Clusters 1 and 3 are the most represented ones (41 and 18 proteins, respectively) and show a linear increase with age, while clusters 2, 4, 5, 6, and 7 (19, 15, 5, 13, and 15 proteins, respectively) show a stepwise decreasing trajectory with age. Most of the proteins in clusters 1 and 3 are related to coagulation and clotting such as fibrinogen, von Willebrand factor, and coagulation factors V and IX.

Among the proteins that are representing the decreasing trajectories, there are several immunoglobulins and serpins (IGLV7‐43, IGLV7‐46, SERPINA4, and SERPINF2 among others). The proteins that represent each cluster are reported in Table S4.

### 2.5 Overlap with SOMASCAN data

To date, large‐scale studies exploring aging and protein expression have employed the SOMASCAN technology (Lehallier et al., 2019; Moaddel et al., 2021; Tanaka et al., 2018). Here, we investigated the overlap in terms of quantified proteins between the SOMASCAN affinity technology‐based Lehallier and the current LC–MS/MS‐based proteomics studies. Based on the protein identifiers mapping to the same genes, we could find 325 genes unique to our study that were not represented in the Lehallier study. A relatively small number (121 genes) was common to both studies indicating the complimentary nature of the two technologies. This analysis is reported in Table S5. It can be noted that about 70 of the unique genes in our study are immunoglobulin genes. The overlap of proteins with significant correlation to age was also examined for the two studies. A total of 15 proteins (reported in Table 2) were found to correlate significantly with age in both studies (q < 0.05).

**TABLE 2:** List of the 15 significant proteins in common between the dataset from this study and the dataset from Lehallier et al.

Protein ID	Protein name	Gene name
O95445	Apolipoprotein M	APOM
P02748	Complement component C9	C9
P10909	Clusterin (Aging‐associated gene 4 protein)	CLU
P54108	Cysteine‐rich secretory protein 3	CRISP3
Q14118	Dystroglycan	DAG1
P23142	Fibulin‐1	FBLN1
Q92820	Gamma‐glutamyl hydrolase	GGH
P80108	Phosphatidylinositol‐glycan‐specific phospholipase D	GPLD1
P69891	Hemoglobin subunit gamma‐1	HBG1
P61626	Lysozyme C	LYZ
P08253	Gelatinase A (Matrix metalloproteinase‐2)	MMP2
Q9H8L6	Multimerin‐2	MMRN2
P00747	Plasminogen	PLG
P08185	Corticosteroid‐binding globulin	SERPINA6
P19320	Vascular cell adhesion protein 1	VCAM1

## 3 DISCUSSION

The results reported in this study show that plasma proteins are differentially expressed across the life span. These differences are displayed in correlation to age, location, and uric acid (UA).

Among the proteins that significantly correlate to age, DAG1 has already been shown to play a crucial role in aging since it has a positive effect on brain homeostasis and neurotrophic activities (Moore et al., 2002; Ohsawa et al. 2001). FBLN1 is a multifunctional extracellular matrix protein (ECM) that binds to many other ECM proteins. In animal models, the absence of the protein leads to vascular anomalies likely related to abnormalities in endothelial cell interactions with subendothelial ECM (Kostka et al., 2001). It is also reported to be involved in the pathophysiology of several kinds of cancers. In bladder cancer, it is epigenetically down‐regulated and functions as a tumor suppressor gene, whereas its overexpression significantly suppresses tumor growth, induces tumor cell apoptosis, decreases cell motility, and inhibits angiogenesis in cultured bladder cancer cells and xenograft tumor in nude mice. In mesothelioma cells, in vitro, FBLN1 also acts as tumor suppressor gene. On the contrary, the expression of FBLN1 is associated with hepatocellular carcinoma (HCC) progression and it is upregulated in the majority of the examined HCC tissues. FBLN1 silencing significantly sensitizes HCC cells to apoptotic signals and decreases the ability of HCC cells to develop tumors in vivo. Serum cell‐free RNA levels are significantly increased in patients with HCC. So, in HCC model it does not work as tumor suppressor gene (Aksoy et al., 2020; Gong et al., 2020; Xiao et al., 2014). Concerning the vascular role of FBLN1, in diabetes or chronic kidney disease increased plasma FBLN1 levels are thought to contribute to thrombotic and cardiovascular complications. The FBLN1 levels fail to demonstrate any effect on vascular age and arterial stiffness in healthy individuals, whereas its down‐regulation seems to be related to aortic dissection (Sang et al., 2021; Scholze et al., 2013; Yasmin et al., 2018). So, in our opinion the increased levels of FBLN1 may simply mirror vascular aging.

We have identified a reduced expression of some immunoglobulin classes and subclasses in LLIs. These data are in partial agreement with previous studies performed with nephelometric assay, which showed an age‐related decrease in IgM and IgG2 (Listì et al., 2006; Lock & Unsworth, 2003). IgG on the whole resulted instead decreased in the present analysis and in the study of Lock and Unsworth but not in that of Listì et al. However, these results represent one aspect of immunosenescence, the reduction in B lymphocytes, which was also previously found in healthy centenarians (Aiello et al., 2019).

Proteins related to inflammation seem to play an important role in our cohort. Interestingly, SOD3 is increasing with age, suggesting a compensatory increase mechanism, since SOD activity has been shown to decrease in LLIs (Belenguer‐Varea et al., 2020). On the contrary, levels of insulin growth factor‐binding proteins, IGFBP‐3 and IGFBP‐5, are reduced in the LLIs. These two proteins have been associated with the induction of cellular senescence (Elzi et al., 2012; Kojima et al., 2012; Sanada et al., 2018). Thus, their decreased levels in healthy aged individuals suggest a possible control of cellular senescence processes, decreasing the inflammation triggered by senescent cells (Tchkonia et al., 2013). On the contrary, studies in models have suggested that old animals were characterized by decreased IGFBP‐3 and IGFBP‐5 in the muscle (Spangenburg et al., 2003). Moreover, in human beings, these proteins have been related to osteoporosis (Ueland et al., 2003). In the largest sample of this cohort, Aiello et al. (2021) showed that the body mass index of centenarians was not significantly different from the values observed in young adults and lower than those observed in older adults. Underweight and overweight conditions are, in fact, considered unfavorable for longevity (Pereira da Silva et al., 2016). However, the data obtained with “bioelectrical impedance analysis” indicate that, on average, the centenarians under study are sarcopenic (Aiello et al., 2021). Regarding bone markers, the larger cohort showed an increase in alkaline phosphatase and osteocalcin with a significant correlation with age and gender. Calcium was significantly decreased in LLI compared with that in young adults and adults. Furthermore, vitamin D levels were significantly reduced in LLI compared with those in the young and adult groups (Aiello et al., 2021). So, IGFBP‐3 and IGFBP‐5 decrease in LLIs could be related to both sarcopenia and osteoporosis.

Lehallier et al. (2019) suggested that a nonlinear approach to study aging would give better interpretation of the biological complexity for this kind of cohorts. For this reason, we have also analyzed our dataset using DEswan in order to identify significant changes in the age waves. Most of the significant proteins in the age waves are unique to LLIs, suggesting a unique protein signature for LLIs. The majority of these significant proteins are related to the extracellular matrix (ECM). The ECM comprises of proteins providing structural and biochemical support to cells. Since the cellular behavior is highly affected by the surrounding environment, ECM age‐related changes impact the ability to give support to the cells and might influence most processes in the body (Birch, 2018; Halper & Kjaer, 2014).

In the present dataset, 29 proteins are significantly correlated to uric acid (UA). UA is the end product of purine metabolism. The role of UA is controversial since it has been reported to enhance oxidative stress (Yu et al., 2010), while other studies suggest that UA is a reactive oxygen species (ROS) scavenger, thus playing an antioxidant effect (El Ridi & Tallima, 2017; Glantzounis et al., 2005; Sautin & Johnson, 2008). Even UA correlation with age is not clear since it has been shown to physiologically increase during adulthood, and it has been associated with endothelial dysfunctions and hypertension (Kawamoto et al., 2013; Kuzuya et al., 2002; Zhou et al., 2017). On the contrary, increased levels of UA in older people have been related to a greater muscle strength (Lee et al., 2019), while in *Caenorhabditis elegans*, UA supplementation increases their life span (Wan et al., 2020). However, UA levels in our whole cohort show no significant differences based on age (Aiello et al., 2021). Among the UA‐related proteins, 9 are represented by cytoskeleton‐associated proteins. These proteins are also increased in older individuals, implying a higher need to recruit proteins to the cytoskeleton to support and maintain cellular homeostasis (Amberg et al., 2012).

Another informative way to explore the data is to look at the protein cluster trajectories. Based on such analysis, most of the proteins in the clusters 1 and 3 in our study are related to coagulation and clotting such as fibrinogen, von Willebrand factor, and coagulation factors V and IX. These proteins are well known to increase with the physiological process of aging and have been reported to be higher in centenarians. This pro‐coagulative state, linked to the fact that many hemostasis factors are inflammatory markers (acute phase proteins), does not involve an increased risk of thrombotic pathologies, as it is controlled by an efficient anti‐inflammatory network (Mannucci et al., 1997; Mari, Coppola, & Provenzano, 2008; Mari et al., 1995).

Among the proteins in the decreasing clusters, serpins were of particular interest. Indeed, serpins are known to be highly secreted by senescent cells, providing a fine balance between thrombosis and thrombolysis cascades, and have been identified as plasma biomarkers of aging (Basisty et al., 2020; Tanaka et al., 2018). The lower levels of serpins strengthen our hypothesis previously suggested when discussing the decrease in IGFBP‐3 and IGFBP‐5, and the possible control of cellular senescence processes in our healthy older and long living individuals (see also above).

Furthermore, when comparing our results with another large study of protein expression across life span (Lehallier et al., 2019), we did identify 15 common proteins between these two datasets. Most of these proteins are known to be involved in aging and/or oxidative stress response, including clusterin, which is known to prevent stress‐induced aggregation of blood plasma proteins (Poon et al., 2000; Wyatt et al., 2009) and matrix metalloprotease 2 (MMP2), reported to be a key player on the inflammatory signaling cascade (Cancemi et al., 2020; Fingleton, 2017) and to be able to reduce inflammation.

## 4 CONCLUSIONS

To conclude, in the present global plasma proteomic study on a cohort of 86 healthy individuals (age range 22–111), we have identified and quantified 410 proteins. Different bioinformatics approaches were performed to investigate proteins that significantly correlate with age, location, and uric acid.

The data on the location, although intriguing, deserve accurate personal data analysis in a new cohort to understand how much they are linked to the place of birth or residence given that many long‐lived individuals born in the villages have moved to the city at various ages.

However, we can formulate the following hypothesis. There are some world zones, called blue zones (BZ), defined as a rather limited and homogenous geographical area (they are essentially endogamous areas, and therefore, their genetic pool is less heterogeneous than that of the rest of the surrounding area) where the population shares the same lifestyle and environment (the different lifestyle and the different environmental exposure are obviously responsible for different epigenetic changes), and their longevity has been proved to be exceptionally high. Two BZs are in the Mediterranean basin, the area of Nuoro in Sardinia and the island of Ikaria, while other areas in Cilento and in Sicani and in Madonie Mountains are considered blue zone‐like because they do not reach the extreme longevity levels of the classic blue zones but they have the same characteristics of geographical and/or historically isolated zones and lifestyle with increased longevity when compared to the surrounding population (Accardi et al., 2019). Accordingly, the Sicani and Madonie populations might have retained significant longevity‐related cause–effect links that in remaining Sicilian population are masked by the effect of greater heterogeneity in genetic, socio‐economic, and lifestyle aspects.

Additionally, we compared our findings with a previously reported study, which also included old individuals. Fifteen proteins were in common between the two studies, confirming that some of the known proteins related to aging were detected in our cohort as well, also suggesting that our approach is able to provide novel information about proteins that are changing across life. Further investigations in separate sample cohorts will be needed to validate the proteins correlating with location and uric acid found in our study, and subsequent functional studies could help to clarify the role of these proteins.

To our knowledge, this is the first untargeted proteomic study performed on plasma of healthy individuals to study healthy aging and longevity. The approach allowed us to identify proteins that could be of biological interest as biomarkers of healthy aging and longevity.

## 5 EXPERIMENTAL PROCEDURES

### 5.1 Study cohort

The participants were recruited from June 2017 to March 2020 within the project “Discovery of molecular and genetic/epigenetic signatures underlying resistance to age‐related diseases and comorbidities (DESIGN, 20157ATSLF),” funded by the Italian Ministry of Education, University and Research. The Ethics Committee of Palermo University Hospital approved the study protocol (Nutrition and Longevity, No. 032017). The study was conducted in accordance with the Declaration of Helsinki and its amendments. All study participants (or their caregivers) gave their written informed consent prior to enrolment.

All study participants were Sicilians, selected on the basis of their health status, and aged between 22 and 111 years. People with chronic invalidating diseases, such as neoplastic and autoimmune ones, as well as with acute disease, such as infectious, and with severe dementia were excluded. To respect privacy, all donors were identified with an alphanumeric code and the data were managed using a database accessible exclusively by researchers involved in the project. A team composed by demographers, biologists and physicians from University of Palermo administered to the participants a detailed questionnaire to collect demographic, clinical, and anamnestic data of interest, as well as functional and cognitive information. The enrolment was conducted using social networks and word of mouth at the University of Palermo for young adults, adults, and older adults, whereas it was conducted at home for the ultranonagenarians. For more information about the recruited population, please see Aiello et al. (2021). A total of 86 healthy donors (females: 53; males: 33; age range 22–111 years) were randomly selected from this large database. Table S1 reports parameters such as gender, age, BMI, category, place of birth, and serum values of uric acid, LDL, HDL, and triglycerides.

### 5.2 Sample preparation for mass spectrometry analysis

Plasma was diluted 10‐fold with 5% SDS in 100 mM Tris (pH = 7.55) and sonicated using a probe sonicator (Branson Digital Sonifier® 250‐D, Branson Ultrasonics Corporation), at an amplitude of 10%, with 10‐s pulse on and 20‐s pulse off, for a total of 36 cycles. The solution was centrifuged at 13,000 rpm for 8 min to remove debris, and the supernatant, containing proteins, was recovered.

A fixed volume (50 μl) of the supernatant, containing approximately 50 μg of plasma proteins, was digested into peptides using on‐bead digestion on HILIC microspheres (ReSyn Biosciences). The process was fully automated using 96‐well plates in King‐Fisher Flex (Thermo Fisher Scientific). The automated procedure consisted of the following steps: magnetic microspheres (1:10 protein: beads ratio) were incubated and equilibrated in equilibration buffer (15% ACN, 100 mM NH4Ac, pH = 4.5); protein sample was incubated in binding buffer (30% ACN, 200 mM NH4Ac, pH = 4.5) where proteins are allowed to bind to HILIC beads. Beads were then washed twice in 95% ACN to remove unspecific and low binding proteins. Bead‐binding proteins were then incubated for 1 h at 47°C with trypsin (20:1 protein: trypsin w/w ratio) dissolved in 50 mM AMBIC. Peptides were recovered from the plate and dried in a SpeedVac (Thermo Fisher Scientific) prior to C18 desalting.

Desalting was performed using BioPureSPN Mini, PROTO 300 C18 (The Nest Group, Inc.). Briefly, columns were equilibrated with 100 μl 70% ACN, 5% FA, and conditioned using 100 μl 5% FA. Samples were resuspended in 100 μl 5% FA and loaded on the column. Columns were washed in 5% FA, and cleaned peptides were eluted using 100 μl 50% ACN, 5% FA. All the steps were performed using an Eppendorf benchtop centrifuge at 50 ***g*** for 2 min.

Cleaned peptides were dried and stored at −20°C prior to quantification and injection into the mass spectrometer.

### 5.3 NanoLC mass spectrometry

Three hundred nanogram peptides (as determined by Nanodrop) of each sample were resuspended in 0.1% FA loaded onto a 1200 EASY‐nano LC system (Thermo Fisher Scientific). The analytical column was 15‐cm‐long fused silica capillary (75 μm × 16 cm Pico Tip Emitter, New Objective) packed in‐house with C18 material ReproSil‐Pur 1.9 μm (Dr. Maisch GmbH). Peptides were separated using an 80‐min method, including a 60‐min linear gradient from 10% to 25% solvent B (80% ACN, 0.1% FA) from 3 to 63 min at a constant flow rate of 250 nl/min. The gradient was preceded by a 3‐min gradient from 5% to 10% B and followed by a 5‐min gradient to 40% B and finally a 5‐min gradient to 90% B followed by 7‐min isocratic washing at 90% B. The nanoLC system was coupled to a Q Exactive HF‐X Mass Spectrometer (Thermo Fisher Scientific). Data were acquired using data‐independent acquisition (DIA).

### 5.4 DIA acquisition

To generate a chromatogram library for DIA processing, the MS was set to acquire six DIA acquisitions with staggered 4 m/z MS/MS spectra (4 m/z precursor isolation windows at 30,000 resolution, AGC target 1e6, maximum inject time 60 ms) using an overlapping window pattern from narrow mass ranges approximately 400–500, 500–600, 600–700, 700–800, 800–900, and 900–1000 m/z, as described in Pino et al. (2020); and with full window MS1 spectra (395–1005 m/z at 30,000 resolution using an AGC target value of 3 × 106 ions and a maximum injection time of 55 ms).

For quantitative samples, the MS was set to acquire full‐scan MS1 spectra as above, and DIA spectra at 15,000 resolution with AGC target of 1 × 10^6^, maximum injection time of 20 ms, loop count 75, and using staggered isolation window of approximately 8 m/z.

DIA spectra were set with normalized collision energy (NCE) of 27. The Xcalibur software v3.0 (Thermo Fisher Scientific) controlled the nanoLC system, and the mass spectrometer was used to acquire and visualize the RAW data.

### 5.5 Mass spectrometry data processing

Raw DIA MS files were converted to mzML using ProteoWizard (Chambers et al., 2012) version 3.0.20079, with vendor peak picking and PRISM demultiplexing. The mzML files were further processed in EncyclopeDIA version 0.9.0 (Searle et al., 2018). A chromatogram library was generated using the pool windows DIA files by matching to a predicted human proteome spectral library (Searle et al., 2020). The spectral library “uniprot_human_25apr2019.fasta.z2_nce33.dlib,” version as of January 28, 2020, was downloaded along with the corresponding UniProt human fasta file from https://www.proteomicsdb.org/prosit. A chromatogram library was generated using default settings in EncyclopeDIA and used for processing of the full DIA acquisitions of the different samples. A quantitative protein group file (1% protein group false discovery rate) for the samples was exported for further analysis. Intensities in the protein file were log2‐transformed and normalized using cyclic LOESS normalization (Gentleman et al., 2005; Smyth, 2005) in NormalyzerDE (Willforss et al., 2019) before further analysis.

### 5.6 Data analysis

Data missingness was analyzed using the “mice” package in R version 4.0.3 (2020‐10‐10). We considered proteins present in at least 50% of the samples for further analyses. Principal component analysis (PCA) was used to perform exploratory analyses of the proteomics data. Explained variance by each principal component was calculated using the output from “prcomp” function in R.

The correlation between principal components and phenotype (clinical) variables was calculated using “lm” function in R, and “adj.r.squared” values were extracted from the summary statistics produced by the “lm” function.

### 5.7 Differential expression analysis

For differential expression analyses, the “limma” R package was used. The following models were used to analyze deferentially abundant proteins:

*Basic model* protein ~ Age.
*Gender and BMI adjusted model* protein ~ gender + BMI + Age.
*Interaction with environmental variables* protein ~ gender + bmi + Age + ENV + Age*ENV.

### 5.8 Age‐related protein clustering

To identify patterns associated with age, we performed cluster analyses of proteins associated with age. To visualize trends, age‐related intervals as following were used:

1. Plot for groups between 0–40, 61–60, 61–80, and 81–111.
2. Plots for groups for age span of 10 years each.

Mean protein intensity was calculated for proteins in each cluster followed by average over each age intervals.

For clustering, protein data were scaled followed by calculating distance between protein observations using Euclidean distance. Clustering was then performed using complete linkage between observations.

### 5.9 Waves of age‐related proteins

The differential expression‐sliding window analysis (DEswan, Lehallier et al., 2019) was used to identify waves of aging plasma proteins.

Age span between 20 and 111 years with interval size of 10 years was selected for sliding window analyses in the DEswan function implemented in the DEswan package. Sex was used as covariate in the DEswan analyses.

## AUTHOR CONTRIBUTIONS

FL, SV, and VS designed the project. VS and SMJ performed the experiment. VS, AA (1), and FL drafted the manuscript. AA (1) performed the statistical analysis. GA, AA (2), MEL, and GC recruited the patients, and collected the samples and all the patient data. CC critically revised the manuscript. All authors have revised and approved the final version of the manuscript.

## CONFLICT OF INTEREST

The authors declare no conflict of interest.

## Supporting information


Figure S1

Figure S2

Table S1

Table S2

Table S3

Table S4

Table S5

## Data Availability

The data that support the findings of this study are available on request from the corresponding author. The data are not publicly available due to privacy or ethical restrictions.
