# Supplementary figures and images for: Plasma proteome profiling of healthy individuals across the life span in a Sicilian cohort with long‐lived individuals
Source: Aging Cell. 2022 Aug 6;21(9):e13684. doi: 10.1111/acel.13684 (PMC9470904; doi:10.1111/acel.13684)

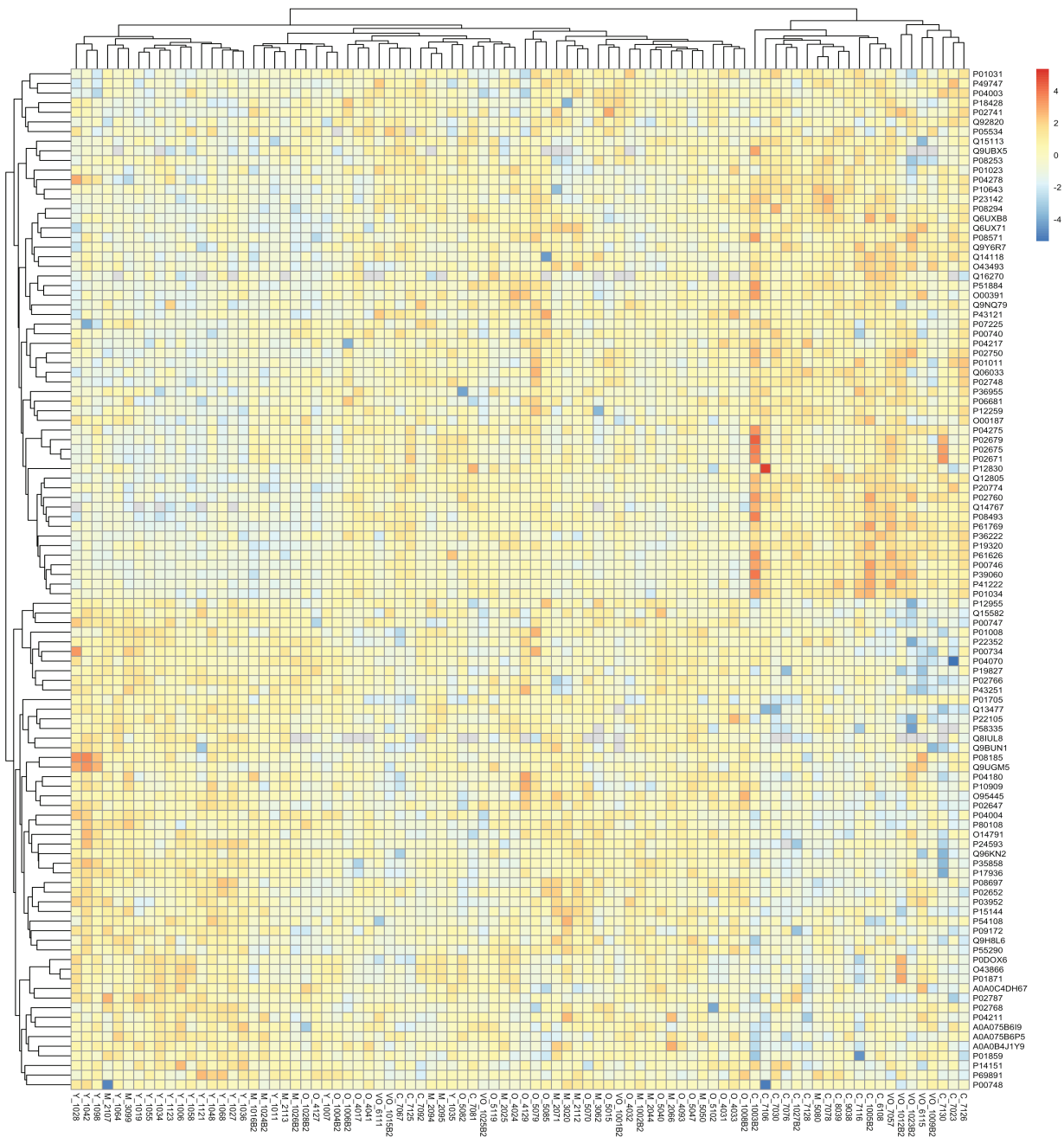

Supplement: Supplementary file 1 — Figure S1 [file ACEL-21-e13684-s003.pdf]
