# Supplementary material for: Plasma proteome profiling of healthy individuals across the life span in a Sicilian cohort with long‐lived individuals
Source: Aging Cell. 2022 Aug 6;21(9):e13684. doi: 10.1111/acel.13684 (PMC9470904; doi:10.1111/acel.13684)
Supplement: Supplementary file 2 — Figure S2 [file ACEL-21-e13684-s002.pdf]

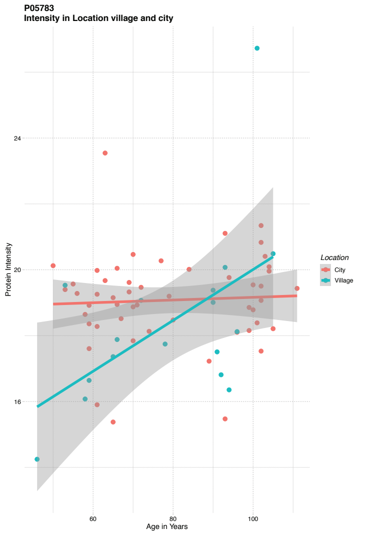
P05783
Intensity in Location village and city
Protein Intensity
24
20
16
60
80
100
Age in Years
Location
City
Village

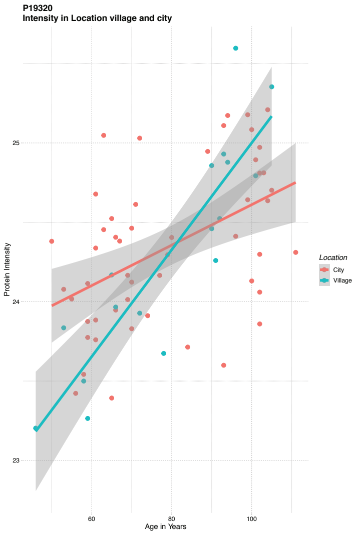
P19320
Intensity in Location village and city
Protein Intensity
25
24
23
60
80
100
Age in Years
Location
City
Village

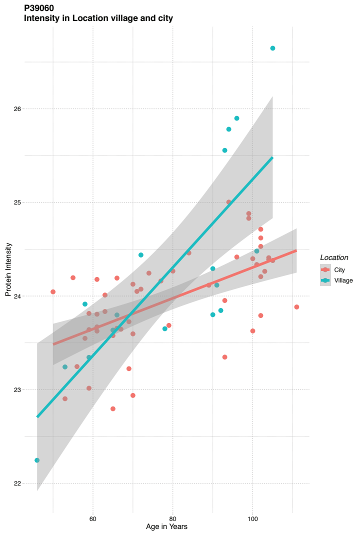
P39060
Intensity in Location village and city
Protein Intensity
26
25
24
23
22
60
80
100
Age in Years
Location
City
Village

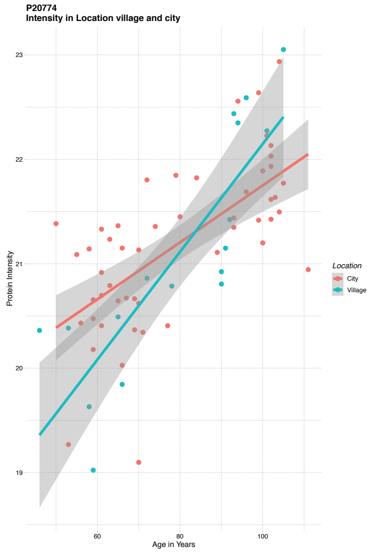
P20774
Intensity in Location village and city
Protein Intensity
23
22
21
20
19
60
80
100
Age in Years
Location
City
Village

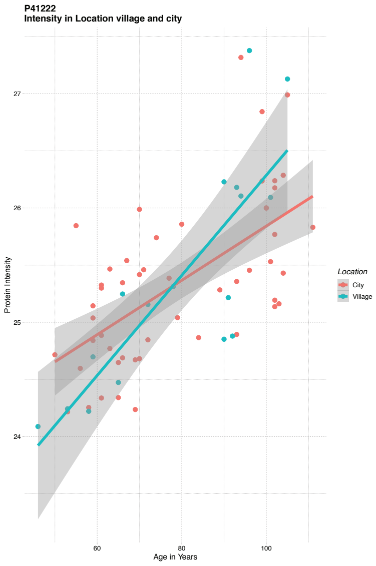
P41222
Intensity in Location village and city
Protein Intensity
27
26
25
24
60
80
100
Age in Years
Location
City
Village

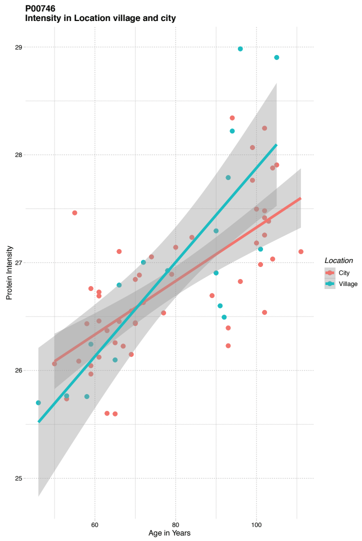
P00746
Intensity in Location village and city
Protein Intensity
29
28
27
26
25
60
80
100
Age in Years
Location
City
Village
